# Keeping hunting bans on target

**DOI:** 10.1111/cobi.13932

**Published:** 2022-06-08

**Authors:** Hugh Webster, Amy Dickman, Adam Hart, Dilys Roe

**Affiliations:** ^1^ Mill Cottage Kirkmichael UK; ^2^ Wildlife Conservation Research Unit, Department of Zoology, Recanati‐Kaplan Centre University of Oxford Oxford UK; ^3^ Department of Natural and Social Sciences University of Gloucestershire Gloucestershire UK; ^4^ IUCN Sustainable Use and Livelihoods Specialist Group London UK

The U.K. government has proposed an Animals Abroad Bill, which would ban the import of hunting trophies. However, this ban is not clearly based on any deontological moral position or system of virtue ethics (Bichel, 2021) because domestic trophy hunting and hunting trophy exports will still be allowed. Nevertheless, the U.K. Government claims its import ban will “disincentivize trophy hunting…and send a positive signal internationally.”

However, if disincentivizing trophy hunting does precipitate a decline in this industry, it may mean fewer jobs, less funding for conservation (antipoaching, etc.), and less local tolerance for wildlife (Naidoo et al., 2016; Parker et al., 2020), all of which would harm human and animal communities. This is why many argue from a consequentialist perspective that trophy hunting bans may inadvertently cause more animal suffering and imperil wildlife rather than save it (Di Minin et al., 2016; Dickman et al., 2019). Indeed, local communities have warned that the proposed ban threatens to “undermine successful community‐based conservation in several African countries” (Communal Areas Management Programme for Indigenous Resources et al., 2021).

Trophy hunting is not listed as a major threat to any species on the International Union for the Conservation of Nature Red List, although it has threatened some specific populations, particularly of lion (*Panthera leo*) and leopard (*Panthera pardus*). But even for these species, this threat is minor compared with threats, such as conflict, habitat loss, and poaching (Bauer et al., 2020; Lindsey, 2015). Indeed, well‐managed hunting actively mitigates these other, greater threats, by protecting habitat and encouraging coexistence, and trophy hunting has demonstrably improved the conservation status of some flagship threatened species (IUCN, 2016; ‘t Sas‐Rolfes et al., 2022).

Some of the community benefits derived from trophy hunting (e.g., meat provision) are also not easily replicated by potential alternatives. In fact, no practicable, scalable, alternative has yet been demonstrated that could similarly sustain the habitat and wildlife protected in hunting zones, while reliably maintaining equivalent community benefits.

Of course, trophy hunting is far from perfect. The industry still exhibits inequitable revenue sharing, occasionally inappropriate or poorly observed quotas, corruption, and inadequate regulation (Lindsey et al., 2007; Loveridge et al., 2007). Like wider tourism, it could benefit from substantive reform. However, the U.K.’s proposed import ban promises to do little to address these problems, targeting good and bad operators alike, while alienating and impoverishing communities that currently generate revenue from sustainable hunting.

Importantly, much land managed for trophy hunting currently generates revenues where phototourism cannot (Lindsey et al., 2007). Hunting, therefore, plays a vital role in protecting less‐favored areas from habitat loss and poaching––threats that pose far greater challenges to threatened species (IUCN, 2016).

The United Kingdom could better support international conservation by designing a smart trophy hunting import ban to incentivize good practice, amplify benefits of sustainable hunting, and encourage reform of remaining bad practice. Such a ban could prohibit import of trophies from canned hunting operations or from operators that fail to demonstrate an equitable sharing of hunting revenues with local communities or establish and monitor sustainable hunting quotas or otherwise fall short of established IUCN principles (IUCN, 2012).

The United States has a similar policy, dropping recently established bans on elephant (*Loxodonta africana*) and lion trophy imports in favor of a case‐by‐case approach that demands trophies originate from sustainable management programs. Similarly, the European Union requires hunting import permits guarantee the origin of a trophy is legal and sustainable.

Legislation aimed at restricting trophy hunting should be based on meaningful consultation with the affected state governments, Indigenous peoples, and local communities and should not undermine successful local conservation (IUCN, 2016). If bans are still deemed necessary, they should only be introduced “after identification and implementation of feasible, fully funded and sustainable alternatives to hunting that respect indigenous and local community rights and livelihoods and deliver equal or greater incentives for conservation” (IUCN, 2016).

The proposed U.K. ban does none of these things; most notably, it fails to provide sustainable or funded alternatives. By contrast, a smart ban focused on promoting sustainability and community rights could encourage improved regulation of the global hunting industry and support development goals and incentivize better conservation.
